# Inflammation and Platelet Activation After COVID-19 Vaccines - Possible Mechanisms Behind Vaccine-Induced Immune Thrombocytopenia and Thrombosis

**DOI:** 10.3389/fimmu.2021.779453

**Published:** 2021-11-23

**Authors:** Sisse R. Ostrowski, Ole S. Søgaard, Martin Tolstrup, Nina B. Stærke, Jens Lundgren, Lars Østergaard, Anne-Mette Hvas

**Affiliations:** ^1^ Department of Clinical Immunology, Copenhagen Hospital Biobank Unit, Rigshospitalet, University of Copenhagen, Copenhagen, Denmark; ^2^ Department of Clinical Medicine, Faculty of Health and Medical Sciences, University of Copenhagen, Copenhagen, Denmark; ^3^ Department Infectious Disease, Aarhus University Hospital, Aarhus, Denmark; ^4^ Department of Clinical Medicine, Aarhus University, Aarhus, Denmark; ^5^ Department of Infectious Diseases, Rigshospitalet, University of Copenhagen, Copenhagen, Denmark; ^6^ Department Clinical Biochemistry, Aarhus University Hospital, Aarhus University, Aarhus, Denmark

**Keywords:** COVID-19, platelet factor 4, thrombocytopenia, thrombosis, vaccines, VITT, TTS

## Abstract

Introduction of vaccines against COVID-19 has provided the most promising chance to control the world-wide COVID-19 pandemic. However, the adenovirus-vector based Oxford/AstraZeneca [ChAdOx1] (AZ) and Johnson & Johnson [Ad26.CoV2.S] COVID-19 vaccines have been linked with serious thromboembolic events combined with thrombocytopenia, denominated Vaccine-induced Immune Thrombocytopenia and Thrombosis (VITT). The pathogenesis of COVID-19 VITT remain incompletely understood; especially the initial events that trigger platelet activation, platelet factor (PF)4 release, complex formation and PF4 antibody production are puzzling. This is a prospective study investigating the impact of different COVID-19 vaccines on inflammation (CRP, TNF-α, IL-1β, IL-6, IL-8, IL-10), vascular endothelial activation (syndecan-1, thrombomodulin, E-selectin, ICAM-1, ICAM-3, VCAM-1), platelet activation (P-selectin, TGF-β, sCD40L) and aggregation (Multiplate^®^ impedance aggregometry), whole blood coagulation (ROTEM^®^), thrombin generation and PF4 antibodies to reveal potential differences between AZ and mRNA vaccines in individuals without VITT. The study included 80 (55 AZ and 55 mRNA) vaccinated individuals and 55 non-vaccinated age- and gender matched healthy controls. The main findings where that both vaccines enhanced inflammation and platelet activation, though AZ vaccination induced a more pronounced increase in several inflammatory and platelet activation markers compared to mRNA vaccination and that post-vaccination thrombin generation was higher following AZ vaccination compared to mRNA vaccination. No difference in neither the PF4 antibody level nor the proportion of individuals with positive PF4 antibodies were observed between the vaccine groups. This is the first study to report enhanced inflammation, platelet activation and thrombin generation following AZ vaccination compared to mRNA vaccination in a head-to-head comparison. We speculate that specific components of the AZ adenovirus vector may serve as initial trigger(s) of (hyper)inflammation, platelet activation and thrombin generation, potentially lowering the threshold for a cascade of events that both trigger complications related to excessive inflammation, platelet and coagulation activation as observed in epidemiological studies and promote development of VITT when combined with high-titer functionally active PF4 antibodies.

## Introduction

Introduction of vaccines against COVID-19 has provided the most promising chance to control the world-wide COVID-19 pandemic. The chimp adenovirus vector-based Oxford/AstraZeneca [AZD1222/ChAdOx1] (AZ) COVID-19 vaccine was approved in the United Kingdom ultimo December 2020 and by the European Medicines Agency at the end of January 2021 and it is one of the most widely used COVID-19 vaccines. In March 2021, the first cases of serious thromboembolic events combined with thrombocytopenia following AZ vaccines were reported (1–3). These events lead to a pause in the use of the AZ vaccine in several European countries, and Denmark later chose to completely remove the AZ vaccine from the general vaccination program. In April 2021, vaccination with another adenovirus vector-based COVID-19 vaccine from Johnson & Johnson [Ad26.CoV2.S] was paused in the United States when similar clinical cases were reported (4–6). Subsequently, larger case series have been published delineating the occurrence and severity of this complication (7–9), now denominated Vaccine-induced Immune Thrombocytopenia and Thrombosis (VITT) (10).

VITT is characterized by multiple thromboses at unusual sites most often as cerebral venous sinus thrombosis (CVST) and splanchnic vein thromboses combined with thrombocytopenia, increased D-dimer and often decreased fibrinogen (1, 2, 7–9, 11). VITT is occasionally complicated by arterial thrombosis as ischemic stroke, myocardial infarction or limb ischemia (3, 12, 13).

The mechanisms behind the pathogenesis of VITT remain incompletely understood, but there is much to suggest that the platelet consumption and the progressive thrombogenic state is initiated by formation of antibodies against the protein platelet factor 4 (PF4). Unusual high serum levels of PF4 antibodies have been found in the vast majority of patients with VITT (1–4, 7, 8). PF4 is a tetrameric chemokine (CXCL4) stored mainly in megakaryocytes and in the α-granules of platelets (14) and released upon platelet activation. In VITT, patients form IgG antibodies against the PF4-polyanion complexes, which activate the platelets *via* the Fcγ receptor on the surface of the platelets. This platelet activation induces increased release of PF4, which contributes to complex formation with the newly formed PF4 autoantibodies leading to thrombocytopenia, increased platelet aggregation and thrombus formation. It is, however, not yet known what triggers the strong autoimmune response in VITT or which components of the vaccines that trigger antibody formation to PF4.

To investigate the impact of COVID-19 vaccines on inflammation and platelets, we analyzed markers of inflammation, vascular endothelial activation, platelet activation and aggregation, whole blood coagulation, thrombin generation and PF4 antibodies pre- and post-vaccination in a cohort of individuals without VITT receiving either AZ or mRNA [Pfizer/BioNTech BNT162b2 and Moderna mRNA-1273] vaccines. For further comparison, an age and gender matched control group comprising healthy non-vaccinated individuals was included.

## Materials and Methods

### Study Participants

Vaccinated study participants were included in the Danish national vaccine trial ENFORCE (*National Cohort Study of Effectiveness and Safety of SARS-CoV-2/Covid-19 vaccines*, https://chip.dk/Research/Studies/ENFORCE/About-ENFORCE) EudraCT no (2020–006003–42). The ENFORCE trial is funded by The Danish Ministry of Health. The ENFORCE trial was approved by the National ethical committee (1-10-72-337-20) and the Danish data protection agency (1-16-02-289-21). Oral and written informed consent from all participants were obtained. The study was conducted in accordance with the declaration of Helsinki.

**Figure 1:**
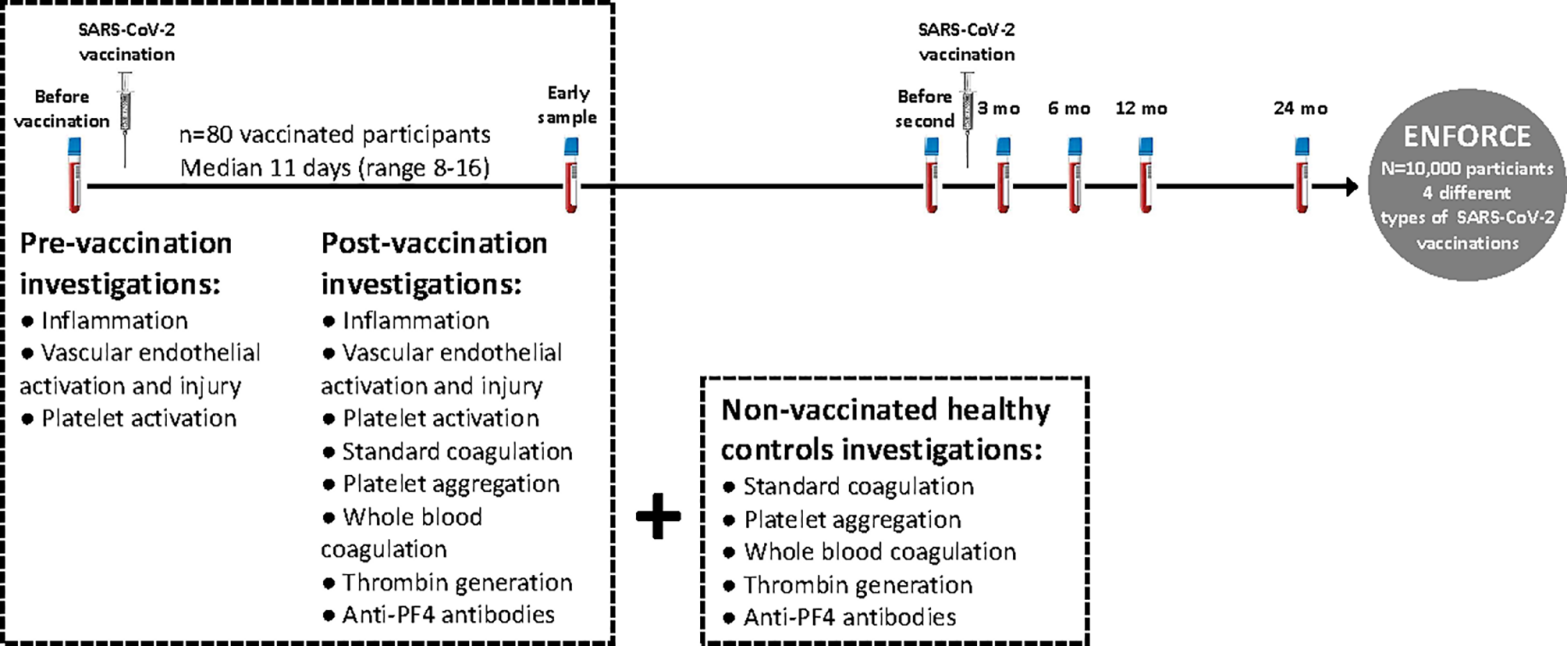
Study design. Eighty participants (n=80) included in the Danish national vaccine trial ENFORCE, recently vaccinated with AZ or mRNA vaccines against COVID-19 were invited to provide an early blood sample post-vaccination (median of 11 days (range 8-16) post-vaccination). Pre-vaccination samples were available from all participants *via* the ENFORCE study. Since standard coagulation tests, platelet aggregation, whole blood coagulation (Thromboelastometry) and thrombin generation were not available at the pre-vaccination sample, non-vaccinated age and gender matched healthy individuals were invited to participate as controls.

Individuals recently vaccinated with either AZ or mRNA vaccines against COVID-19 were invited to provide an early post-vaccination blood sample (see **Figure 1** for study design). The present study included eighty participants recently vaccinated (median of 11 days (range 8-16) post-vaccination) with either AZ (n=55, Oxford/AstraZenica [AZD1222/ChAdOx1]) or mRNA (n=25 in total: n=16 Pfizer/BioNTech [BNT162b2] and n=9 Moderna [mRNA-1273]) vaccines. Pre-vaccination samples were available from all participants, as they were participants in the ENFORCE study.

Since blood samples for platelet aggregation, whole blood coagulation and thrombin generation were not available at the pre-vaccination sample, non-vaccinated healthy individuals (controls) were invited to participate. The non-vaccinated controls were included in a separate study approved by the regional ethical committee (1-10-72-208-21) and the data protection agency (1-16-02-246-21). Oral and written informed consent from all participants were obtained. The study was conducted in accordance with the declaration of Helsinki. Eighty controls volunteered to participate and among these, we selected a subgroup that was age matched (age distribution matched from 20-70+ years for each 10-year interval with random and data blinded removal of individuals within age intervals with overrepresentation) and gender matched (similar approach as for the age matching) with the entire vaccination group (n=80). The age and gender matched controls comprised 55 individuals.

### Participant Data

Age, gender and information about anticoagulant medication or platelet inhibitors were registered upon study participation and blood sampling.

### Biomarkers of Inflammation, Vascular Endothelial Activation and Platelet Activation

Biomarker analysis was conducted pre- and post-vaccination. Markers reflecting inflammation (C-reactive protein (CRP), tumor necrosis factor (TNF)-α, interleukin (IL)-1β, IL-6, IL-8, IL-10), vascular endothelial activation (syndecan-1, thrombomodulin, endothelial (E)-Selectin, intercellular adhesion molecule (ICAM)-1, ICAM-3, vascular cell adhesion molecule (VCAM)-1) and platelet activation (platelet (P)-Selectin, transforming growth factor (TGF)-β, soluble CD40 ligand (CD40L)) were measured in EDTA plasma by MesoScale Discovery (MSD) single or multiplex panels according to the manufacturer’s recommendation by a MESO QuickPlex SQ 120 instrument (Rockville, Maryland, US). The following panels were applied: Proinflammatory panel I (TNF-α, IL-1β, IL-6, IL-8, IL-10). Human vascular injury kit I (E-Selectin, ICAM-3, P-Selectin, Thrombomodulin). Human vascular injury (CRP, ICAM-1, VCAM-1). Human syndecan-1 assay (syndecan-1). Human effector checkpoint assay (soluble CD40L). Human TGF-β1 assay (TGF-β). Results are displayed as concentrations (either pg/ml, ng/ml or µg/ml as appropriate).

### Standard Coagulation, Platelet Aggregation (Multiplate), Whole Blood Coagulation (Thromboelastometry, ROTEM) and Thrombin Generation

All analyses were performed post-vaccination and in non-vaccinated healthy controls in an ISO 15189 accredited laboratory.

#### Standard Coagulation

In brief, platelet count was measured in EDTA plasma employing Sysmex XN-9000 (Sysmex, Kobe, Japan). Blood samples for the remaining coagulation tests were obtained using 3.2% sodium citrate. Fibrinogen (functional, Clauss methods), D-dimer, international normalized ratio (INR), and activated partial thrombin time (aPTT) were analyzed employing Sysmex C5100 (Sysmex, Kobe, Japan).

#### Platelet Aggregation

Blood was collected in tubes anticoagulated by hirudin. The analysis was performed within 2 hours employing impedance aggregometry (Multiplate^®^ Analyzer, Roche, Germany). Platelets were activated with adenosine diphosphate (ADP) (Roche ADPtest), collagen (Bio/Data Corporation, BioNordika, Herlev, Denmark), and arachidonic acid (AA) (Roche ASPItest), according to the manufacturer’s recommendations. The area under curve (AUC, aggregation units* min) was registered.

#### Whole Blood Coagulation

Whole blood for thromboelastometry (ROTEM, Instrumentation Laboratory, Bedford, USA) was collected using 3.2% sodium citrate tubes. The samples were left to rest for 30 min and analyzed within 2 hours. The standard assays EXTEM, INTEM, and FIBTEM were performed according to the manufacturer’s recommendations. The following parameters were registered: clotting time (CT, seconds), maximum clot formation (MCF, mm) and lysis at 30 minutes (LI30, %).

#### Thrombin Generation

Blood was collected using sodium citrate 3.2% tubes, centrifuged at 3,000 g for 25 min within 1 hour after blood sampling to obtain platelet-poor plasma, aliquoted, and stored at -80°C until analysis. The thrombin generation assay (calibrated automated thrombogram^®^, Thrombinoscope^®^ BV, Maastricht, the Netherlands) was performed as previously described, with final concentration of 5 pM tissue factor in wells (15). The following parameters were registered: Lag time (min), time to peak thrombin concentration (ttPeak, min), peak thrombin concentration (peak, nM), and endogenous thrombin potential (ETP, area under curve, nM*min).

### Platelet Factor (PF)4 Antibodies

PF4 antibodies were investigated post-vaccination and in non-vaccinated healthy controls.

The immunoglobulin-G (IgG) specific ELISA-based assay Lifecodes PF4 IgG (Immucor, Solihull, United Kingdom) was employed to measure antibodies against PF4 (16). The blood samples were collected in tubes without anticoagulant, and serum was separated from red blood cells within 2 hours and stored at -80°C until analysis. The analysis was performed in duplicate and in case of more than 20% deviation the test was re-run. Based on 120 serum samples from healthy individuals, the manufacturer calculated a 95% reference interval with a 90% confidence and stated the upper end of the reference interval to be 0.352 O.D. units. According to the manufacturer, test results showing O.D. values equal to or greater than 0.400 are regarded as positive results.

### Statistics

Data on demography, biomarkers, aggregation, whole blood coagulation, thrombin generation and PF4 antibodies are displayed as medians with interquartile ranges (IQR) or as n and proportions. AZ and mRNA vaccinated individuals (pre-, post-vaccination, delta values) and non-vaccinated controls were compared by Mann-Whitney U tests or Chi-square/Fishers exact tests, as appropriate. Delta values were calculated as post-vaccination values minus pre-vaccination values. Changes in variables over time (from pre- to post-vaccination) within sub-groups (AZ and mRNA vaccinated) were compared by Wilcoxon singed-rank test. Spearman correlations were conducted to investigate correlations between platelet count post-vaccination and platelet activation biomarkers and to investigate the intercorrelation between platelet activation biomarkers, results are displayed as rho and p-values. IBM SPSS statistics v. 25 were used for all analyses and figures. P-values <0.050 are considered significant.

## Results

### Study Participants

Eighty vaccinated were included; 55 vaccinated with AZ and 25 vaccinated with mRNA COVID-19 vaccines. The median age of the entire group (AZ + mRNA) was 49 years (IQR 37-58), 73% were women. The AZ and mRNA groups had comparable age (median 48 (IQR 35-57) *vs* 49 (IQR 38-61) years), but the AZ group comprised a higher proportion of women (n=44, 80%) than the mRNA group (n=14, 56%) (p=0.026). It should be emphasized that based on the vaccination program in Denmark, the mRNA vaccinated individuals were more vulnerable and had a higher degree of co-morbidity compared to the AZ vaccinated individuals, as the latter mainly comprised healthcare professionals. This “vaccine group bias” may also explain the higher proportion of women in the AZ group compared to the mRNA group.

Among the vaccinated individuals, four subjects (two in each group) received aspirin (ASA). One individual in the mRNA group received a direct oral anticoagulant drug and one individual in the AZ group received a vitamin K antagonist.

Fifty-five non-vaccinated age and gender matched healthy individuals (controls) were included (median age 49 years (IQR 38-56), 73% women). We considered the matching successful, as median age and gender composition in the controls were comparable to both the entire cohort, the AZ and the mRNA groups (all p=NS).

### Inflammation and Vascular Endothelial Activation

#### Pre-Vaccination

**Figure 2:**
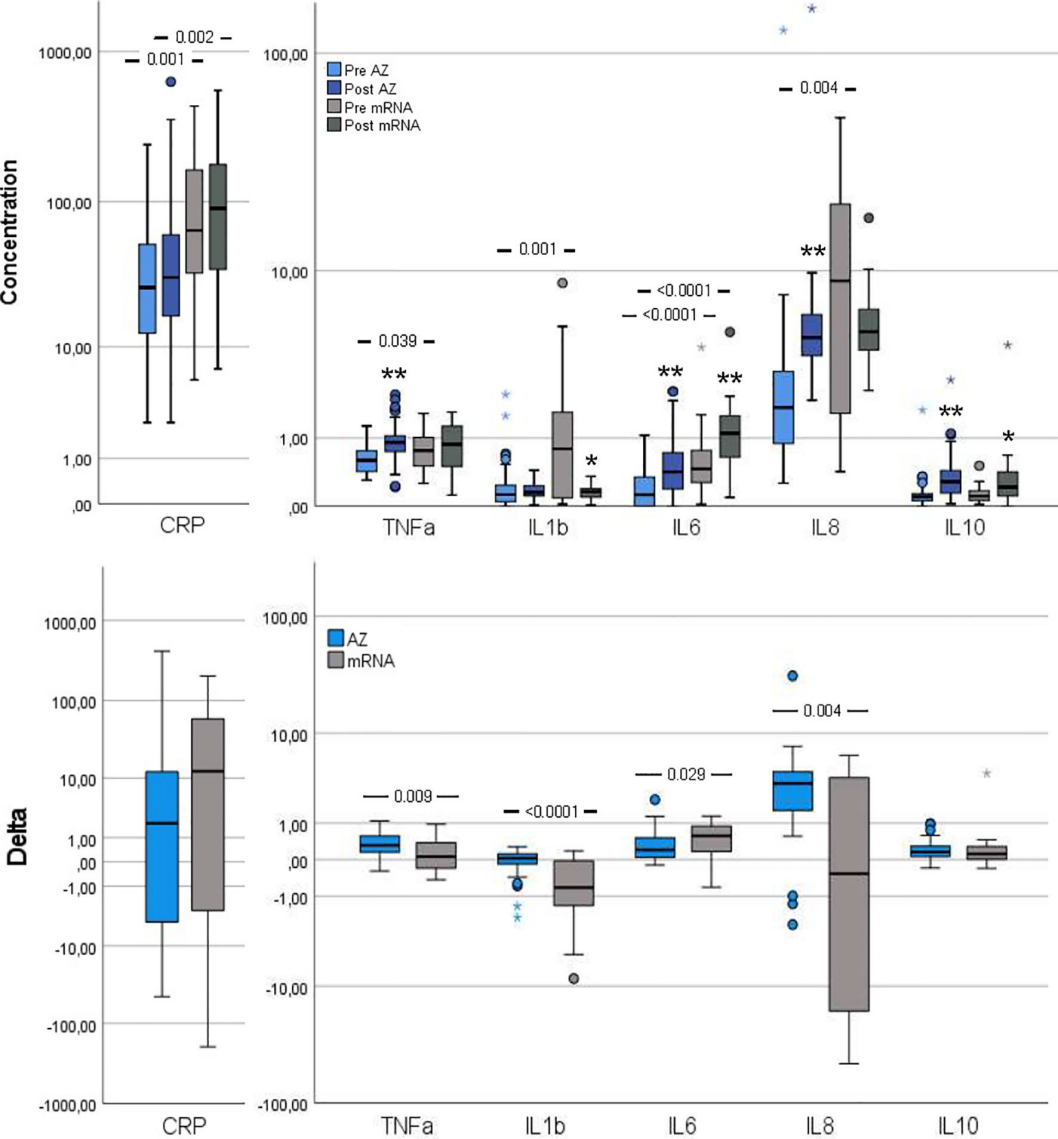
Inflammation markers pre- and post-vaccination and delta changes with either AZ (n=55) or mRNA (n=25) vaccines. Levels of C-reactive protein (CRP, µg/ml), Tumor necrosis factor (TNF)-α (pg/ml), interleukin (IL)-1β (pg/ml), IL-6 (pg/ml), IL-8 (pg/ml) and IL-10 (pg/ml) are displayed on log scales. The top figure displays pre- and post-vaccination values, and the bottom figure displays delta changes. The box plots display median, first and third quartile (box mid-line, button and top, respectively) and the whiskers display maximum and minimum values with outliers (circles) and extremes (small asterisks) displayed. AZ vaccinated individuals are displayed by blue bars (top figure: pre=light blue, post=dark blue) and mRNA vaccinated individuals are displayed by gray bars (top figure: pre = light gray, post=dark gray). Differences between AZ and mRNA vaccinated individuals either pre- and post-vaccination and delta changes are investigated by Mann-Whitney U test, with p-values displayed. Changes over time within the AZ or mRNA groups are investigated by Wilcoxon signed-rank test, with **p < 0.0001 and *p < 0.010 (displayed over the post-vaccination bars).

**Figure 3:**
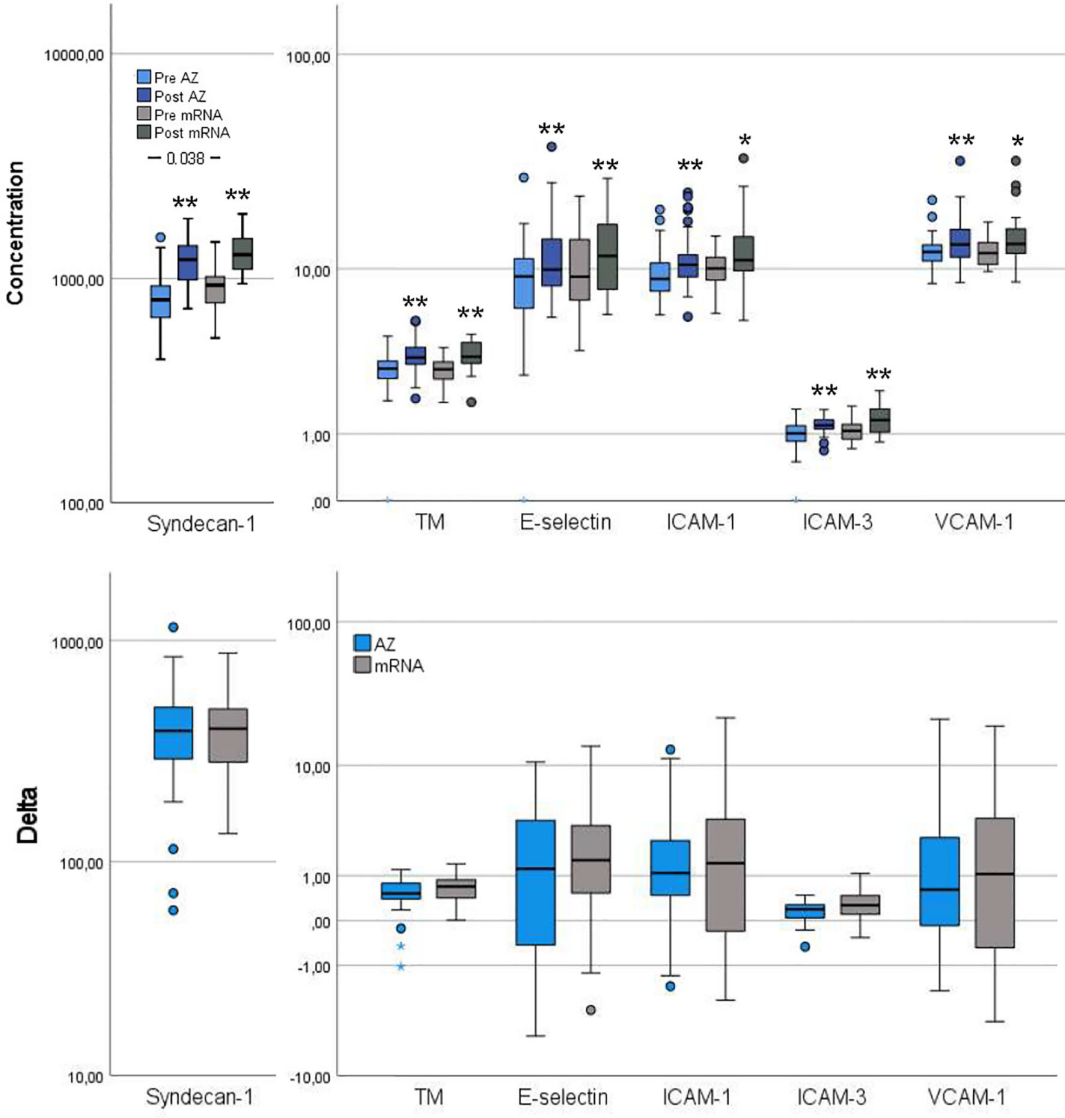
Vascular endothelial activation markers pre- and post-vaccination and delta changes with either AZ (n=55) or mRNA (n=25) vaccines. Levels of Syndecan-1 (pg/ml), thrombomodulin (TM, ng/ml), endothelial (E)-selectin (ng/ml), intercellular adhesion molecule (ICAM)-1 (µg/ml), ICAM-3 (ng/ml) and vascular cell adhesion molecule (VCAM)-1 (µg/ml) are displayed on log scales. The top figure displays pre- and post-vaccination values, and the bottom figure displays delta changes. The box plots display median, first and third quartile (box mid-line, button and top, respectively) and the whiskers display maximum and minimum values with outliers (circles) and extremes (small asterisks) displayed. AZ vaccinated individuals are displayed by blue bars (top figure: pre=light blue, post=dark blue) and mRNA vaccinated individuals are displayed by gray bars (top figure: pre=light gray, post=dark gray). Differences between AZ and mRNA vaccinated individuals either pre- and post-vaccination and delta changes are investigated by Mann-Whitney U test, with p-values displayed. Changes over time within the AZ or mRNA groups are investigated by Wilcoxon signed-rank test, with **p < 0.0001 and *p < 0.010 (displayed over the post-vaccination bars).

Several inflammation markers were higher in the mRNA group compared to the AZ group: CRP, TNF-α, IL-1β, IL-6 and IL-8 (**Figure 2**). Furthermore, pre-vaccination, syndecan-1, a vascular endothelial glycocalyx marker, was increased in the mRNA group whereas the remaining vascular endothelial markers were comparable between groups (**Figure 3**).

#### Post-Vaccination

CRP and IL-6 remained higher in the mRNA group whereas TNF-α, IL-1β and IL-8 changed in magnitude, so the AZ and mRNA groups had comparable levels (**Figure 2**). IL-10 neither differed between groups pre- nor post-vaccination. Post-vaccination, TNF-α and IL-8 only increased in the AZ group whereas IL-6 and IL-10 increased in both groups. CRP did not change in either group and IL-1β did not change in the AZ group whereas it declined in the mRNA group. Post-vaccination, no differences between groups were observed in any vascular endothelial markers and the increase in these (delta values) was comparable (**Figure 3**).

#### Delta Changes

The delta increases in TNF-α, IL-1β and IL-8 were higher in the AZ group whereas the increase in IL-6 was higher in the mRNA group (**Figure 2**). The delta change in CRP and IL-10 did not differ between groups.

### Platelet Activation

#### Pre-Vaccination

P-selectin and CD40L were both higher in the mRNA group than in the AZ group whereas TGF-β did not differ between groups.

#### Post-Vaccination

**Figure 4:**
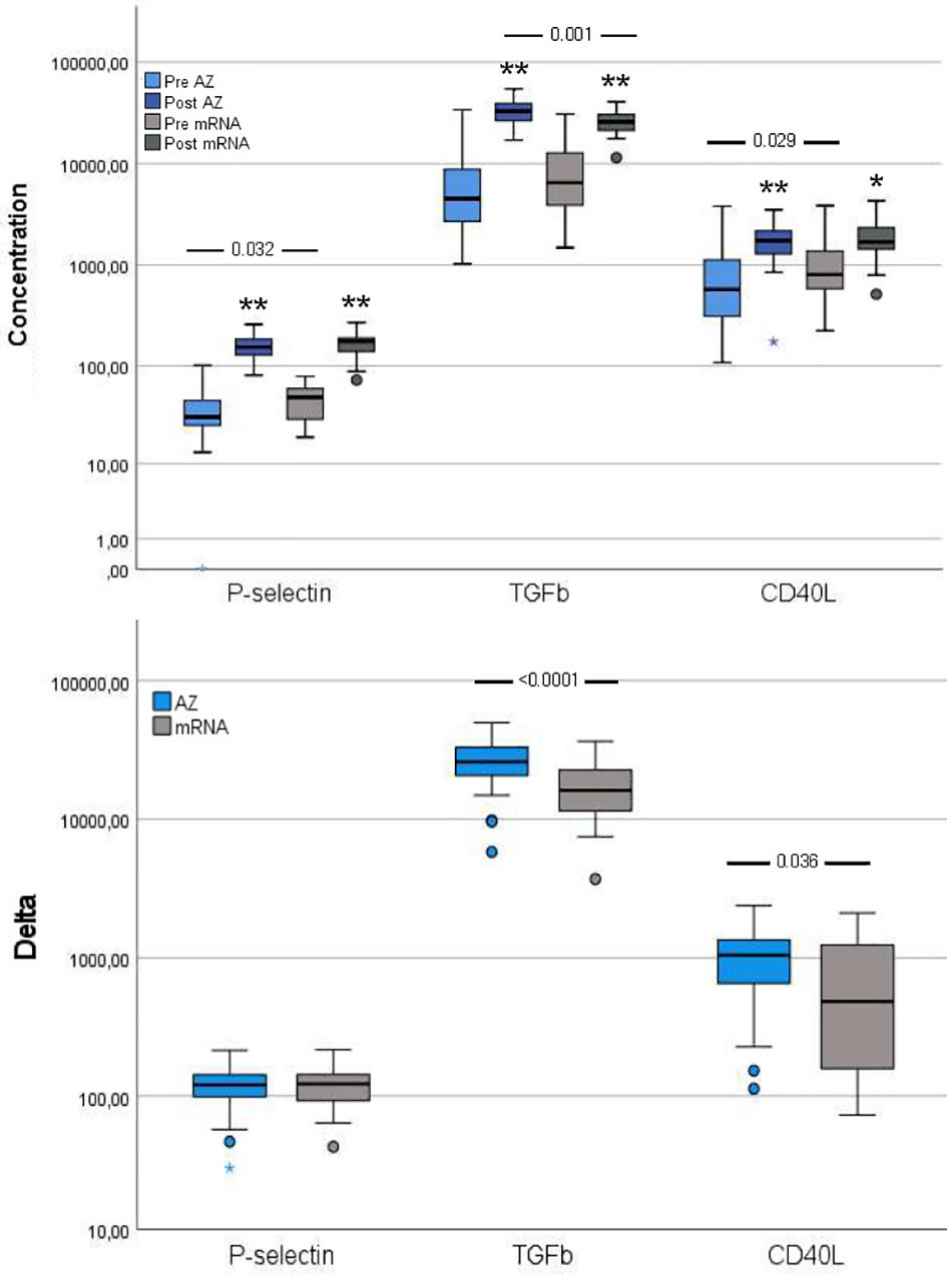
Platelet activation markers pre- and post-vaccination and delta changes with either AZ (n=55) or mRNA (n=25) vaccines. Levels of platelet (P)-selectin (ng/ml), transforming growth factor (TGF)-β (pg/ml) and soluble CD40 ligand (CD40L, pg/ml) are displayed on a log scale. The top figure displays pre- and post-vaccination values, and the bottom figure displays delta changes. The box plots display median, first and third quartile (box mid-line, button and top, respectively) and the whiskers display maximum and minimum values with outliers (circles) and extremes (small asterisks) displayed. AZ vaccinated individuals are displayed by blue bars (top figure: pre=light blue, post=dark blue) and mRNA vaccinated individuals are displayed by gray bars (top figure: pre=light gray, post=dark gray). Differences between AZ and mRNA vaccinated individuals either pre- and post-vaccination and delta changes are investigated by Mann-Whitney U test, with p-values displayed. Changes over time within the AZ or mRNA groups are investigated by Wilcoxon signed-rank test, with **p < 0.0001 and *p < 0.010 (displayed over the post-vaccination bars).

Though the three platelet activation markers increased from pre- to post-vaccination in both groups, compared to pre-vaccination, a different pattern was observed post-vaccination, with higher TGF-β in the AZ group and comparable levels of P-selectin and CD40L between the vaccination groups (**Figure 4**).

#### Delta Changes

The delta increase in TGF-β and CD40L were higher in the AZ group compared to the mRNA group (**Figure 4**).

Since the investigated platelet activation biomarkers, are also released to a lesser extend from other cells than platelets, we investigated the intercorrelations between the investigated platelet activation markers (**Table S1**) and between post-vaccination platelet count and platelet activation markers (**Figure S1**).

In brief, all platelet activation markers were intercorrelated, with the strongest being between TGF-β and CD40L (pre-vaccination: rho=0.93-0.94; post-vaccination and delta: rho=0.69-0.90). P-selectin generally correlated weaker with the other platelet activation markers. Despite the mRNA group being the smallest, some of the strongest intercorrelations were observed in this group post-vaccination and for delta values (**Table S1**).

Post-vaccination platelet count correlated generally with both pre- and post-vaccination platelet activation markers, with the strongest correlations observed between post-vaccination platelet count and post-vaccination TGF-β (rho=0.56-0.60) followed by CD40L (rho=0.37-0.42). P-selectin only correlated weakly with platelet count (**Figure S1**).

### Standard Coagulation, Platelet Aggregation, Whole Blood Coagulation and Thrombin Generation

We only had data on standard coagulation, platelet aggregation, whole blood coagulation and thrombin generation post-vaccination due to the design of the study (**Figure 1**).

Post-vaccination, the AZ group had higher platelet count than the mRNA group whereas the mRNA group had higher INR, fibrinogen and higher INTEM LI30 indicating less fibrinolysis (**Table 1**). The AZ group had shorter lagtime and ttPeak and higher Peak and ETP than the mRNA group, all parameters indicating higher thrombin generation in the AZ group (**Table 1**).

**Table 1:** Coagulation, primary and secondary hemostasis and thrombin generation post-vaccination either AZ (n=55) or mRNA (n=25) vaccines and in non-vaccinated age and gender matched controls (n=55).

Group	Unit	AZ	mRNA	AZ *vs *mRNA	Controls	Con *vs* AZ	Con *vs* mRNA
		Median (IQR)	Median (IQR)	p-value	Median (IQR)		
**Standard coagulation**							
Platelet count	10^9^/L	285 (251-329)	265 (217-279)	**0.013**	245 (215-281)	**<0.0001**	NS
D-dimer	FEU/L	0.30 (0.25-0.43)	0.26 (0.25-0.38)	NS	0.25 (0.25-0.32)	**0.004**	NS
Fibrinogen	µmol/L	8.78 (7.39-9.92)	10.59 (8.45-12.54)	**0.004**	8.29 (7.08-9.71)	NS	**0.001**
aPTT	Sec	23.3 (21.8-25)	22.6 (21.7-24.4)	NS	23.7 (22.7-24.7)	NS	**0.032**
INR	IU	1.00 (1.00-1.06)	1.00 (1.00-1.00)	NS	1.00 (1.00-1.05)	NS	**0.042**
**Platelet aggregation (Multiplate)**							
COLtest	AUC	688 (582-770)	695 (645-766)	NS	464 (367-611)	**<0.0001**	**<0.0001**
ADPtest	AUC	809 (690-984)	808 (677-964)	NS	810 (754-1,007)	NS	NS
ASPItest	AUC	1,060 (896-1,194)	1,066 (791-1,164)	NS	1,074 (962-1,164)	NS	NS
ASPItest*	AUC	1,065 (946-1,206)	1,066 (800-1,175)	NS	1,074 (962-1,164)	NS	NS
**Thromboelastometry (ROTEM)**							
EXTEM CT	Sec	62 (60-67)	64 (58-66)	NS	63 (56-67)	NS	NS
EXTEM MCF	mm	65 (62-67)	66 (62-68)	NS	63 (60-66)	**0.007**	**0.023**
EXTEM LI30	%	100 (100-100)	100 (100-100)	NS	100 (100-100)	NS	NS
INTEM CT	Sec	187 (178-198)	184 (175-212)	NS	181 (173-190)	**0.015**	NS
INTEM MCF	mm	63 (62-66)	64 (61-67)	NS	63 (60-65)	NS	NS
INTEM LI30	%	99 (98-100)	100 (99-100)	**0.007**	100 (99-100)	**0.007**	NS
FIBTEM MCF	mm	16 (13-19)	16 (14-20)	NS	15 (11-17)	**0.007**	**0.014**
**Thrombin generation**							
Lagtime	min	2.74 (2.40-3.40)	3.33 (3.00-3.33)	**0.011**	2.83 (2.67-3.00)	**NS**	**<0.0001**
Peak	nM	241 (198-279)	198 (174-253)	**0.030**	221 (190-254)	NS	NS
ttPeak	min	6.24 (5.41-7.24)	7.00 (6.50-7.71)	**0.003**	6.33 (6.00-7.00)	NS	**<0.0001**
ETP	nM*min	1,430 (1,346-1,673)	1,318 (1,195-1,612)	**0.050**	1,360 (1,270-1,492)	**0.015**	NS

Platelet count normal range: Females: 165-400; Males: 145-350. D-dimer normal range: <50 years <0.5 FEU/L; >50 years <0.6 FEU/L. *Two individuals in each group received ASA, median (IQR) values are displayed without these individuals included. Post-vaccination values in AZ and mRNA vaccinated individuals are compared by Mann-Whitney U test. P-values <0.050 are displayed in bold. P-values >0.10 are displayed as NS (not significant). aPTT, activate partial thrombin time; INR, international normalized ratio; CT, clotting time. MCF, maximum clot firmness; LI30, lysis after 30 min; ttPeak, time to peak; ETP, endogenous thrombin potential.

Compared to non-vaccinated controls, the AZ group had higher platelet count and higher D-dimer post-vaccination whereas the mRNA group had higher fibrinogen and lower aPTT and INR (**Table 1**). Furthermore, both vaccination groups had higher post-vaccination platelet aggregation indicated by higher COLtest aggregation, and stronger clot formation indicated by higher EXTEM MCF and FIBTEM MCF than non-vaccinated controls (**Table 1**). Finally, the AZ group had longer post-vaccination INTEM CT, lower INTEM LI30 (indicating more fibrinolysis) and higher ETP (indicating more thrombin generation) than controls whereas, the mRNA group had longer lagtime and ttPeak than controls (**Table 1**).

### Platelet Factor (PF)4 Antibodies

PF4 antibodies were analyzed post-vaccination and in controls. Post-vaccination, one individual in the mRNA group and two individuals in the control group had positive PF4 antibodies (>0.400 O.D.) whereas no individuals had O.D. >0.400 in the AZ group (p=NS). The post-vaccination median level of PF4 antibodies in the AZ and mRNA groups did not differ (0.11 O.D. (IQR 0.08-0.16) *vs*. 0.09 O.D. (IQR 0.07-0.11), p=NS). The PF4 antibody level in controls was 0.09 O.D. (IQR 0.06-0.13), which was lower than the AZ group post-vaccination (p=0.021) but comparable to the mRNA group post-vaccination (p=NS).

## Discussion

The main finding in the present study was that AZ vaccination against COVID-19 induced a more pronounced increase in several inflammatory and platelet activation markers compared to mRNA vaccination. Also, post-vaccination thrombin generation was higher for all parameters in AZ vaccinated individuals than in mRNA vaccinated individuals. We found no difference in neither the PF4 antibody level nor the proportion of PF4 antibody positives between the vaccination groups.

The present study investigated individuals vaccinated against COVID-19 with either AZ or mRNA vaccines. None of the study participants developed VITT so our findings reflect normal vaccination responses to the investigated vaccines. The finding that AZ vaccination induced enhanced inflammation, platelet activation and thrombin generation compared to mRNA vaccination in a head-to-head comparison is notable and has to our knowledge not previously been reported. Importantly, our finding is in accordance with that of a recent epidemiologic study, reporting that AZ vaccination is associated with an increase in venous thromboembolic events, including CVST, and slightly higher rates of thrombocytopenia, coagulation disorders and bleeding (17). Together this indicates that even in a population with normal vaccination responses, higher levels of complications related to excessive inflammation, platelet and coagulation activation are observed following AZ vaccination.

Vaccines are designed and administered to inflict an immune response (18, 19), and some degree of inflammation and platelet activation is to be expected post-vaccination [the latter due to the close link between innate immunity and platelet activation (20)], in accordance with the findings in the present study that both vaccines resulted in enhanced inflammation and platelet activation one week after vaccination. Despite this common vaccination response, it is however notable, that the AZ vaccine induced higher increases in several key inflammatory mediators (TNF-α, IL-1β and IL-8) compared to mRNA vaccines, also given that the mRNA vaccinated individuals had a higher degree of low-grade inflammation pre-vaccination.

Though the mechanisms behind the pathogenesis of VITT remain incompletely understood, the initial events that trigger platelet activation, PF4 release, complex formation and PF4 antibody production (whichever comes first initially) are especially puzzling. It has been reported that PF4 antibodies are present at low levels in up to 7% of healthy individuals (21) and that healthy individuals have a reservoir of B cells specific for PF4 complexes, but that different immunoregulatory mechanisms hold these B cells under control (22, 23). It has also been reported that almost 7% of COVID-19 vaccinated individuals (both adenovirus vector-based vaccines and mRNA vaccines) have low titers of PF4 antibodies, but these antibodies are not functionally active (24). Given that PF4 in healthy individuals is stored (hidden) in the megakaryocytes and in the α-granules of platelets (14), an early event post-vaccination leading to a breach of tolerance and generation of functionally active PF4 antibodies would be necessary to induce ensuing platelet activation and release of PF4 (25). Several studies have focused on vaccine components from the AZ adenovirus vector vaccine as a trigger of the cascade of early events (1, 25, 26). Though no specific component(s) have yet been identified as trigger of VITT, it has been suggested that one critical downstream event may be hyperinflammation induced by the AZ adenovirus vector vaccine (25). This notion is supported by our finding of higher increases in inflammation following the AZ vaccine compared to mRNA vaccines and the finding of higher increases in the chemokine IL-8/CXCL8, a potent neutrophil chemoattractant and activator (27, 28), is notable given that neutrophil activation and NETosis have been proposed to be involved in VITT pathophysiology (25). This study can only speculate on mechanisms contributing to the observed (hyper)inflammation observed following the AZ vaccine and further studies are needed to reveal potential structural components of the Adenovirus vector vaccines that may contribute to the (hyper)inflammation observed in this and other studies (1, 25, 26).

The AZ and mRNA vaccines both enhanced inflammation, platelet activation and vascular endothelial activation, a notable finding given that COVID-19 itself is also associated with extensive inflammation and platelet activation with e.g. high circulating proinflammatory cytokine levels, enhanced P-selectin expression and high soluble levels and extensive widespread vascular endothelial activation (29–33). Several studies of COVID-19 associated platelet activation or vascular endothelial activation have included advanced *OMICS* analyses, and despite the few biomarkers in common with our study, it is difficult to directly compare the COVID-19 vaccine and SARS-CoV-2 infection/COVID-19 induced changes. Though it is not possible from the design of the present study, to determine to which degree the observed platelet and vascular endothelial activation observed following COVID-19 vaccines is attributed to direct effects of SARS-CoV-2 antigens or indirect effects due to (hyper)inflammation, we envision that (hyper)inflammation is the main driver following vaccination whereas SARS-CoV-2 antigens (viremia) may be a greater contributor in COVID-19. Despite the common platelet activation following different COVID-19 vaccines, the present study also found higher increases in platelet activation and enhanced thrombin generation following the AZ vaccine compared to mRNA vaccines. This is to our knowledge the first study reporting this based on a head-to-head comparison. Given that platelet activation is a hallmark of VITT, the finding of higher increases in biomarkers suggestive of platelet degranulation combined with enhanced thrombin generation following the AZ vaccine is highly intriguing. Though VITT (34, 35) and CVST (9) have been reported following COVID-19 vaccination with mRNA vaccines, only CVST following AZ vaccination is associated with thrombocytopenia (9), emphasizing the critical involvement of platelets in VITT and potentially also the cascade of events that trigger VITT. We speculate that specific components of the AZ adenovirus vector may serve as initial trigger(s) of (hyper)inflammation, platelet activation and thrombin generation, potentially lowering the threshold for a cascade of events that both trigger complications related to excessive inflammation, platelet and coagulation activation as observed in epidemiologic studies (17) and promote development of VITT when combined with high-titer functionally active PF4 antibodies.

Finally, the present study found no evidence of enhanced PF4 antibody production following AZ vaccination compared to mRNA vaccination. This finding is not surprising given the previous finding from larger and better powered studies investigating PF4 antibodies in AZ vaccinated individuals (24, 36). The finding however emphasizes that the observed higher increase in platelet activation and thrombin generation in AZ vaccinated individuals compared to mRNA vaccinated individuals is not dependent only of PF4 antibodies. This points to specific components in the AZ adenovirus vector vaccine as initial trigger(s) of (hyper)inflammation, platelet activation and thrombin generation.

The study had several limitations. The study included a low number of participants and conducted many different investigations, together increasing the risk of both Type I and Type II errors. This should be taken into account, when interpreting the results (p-values just below 0.050 may be by chance). Furthermore, for the whole blood coagulation tests, we did not have pre-vaccination values for comparison but instead had to include an age and gender matched control group for these comparisons. Finally, we did not collect a full medical history on the vaccinated study participants, despite inferring that the vaccination program in Denmark introduced a “vaccine group bias” as mainly vulnerable individuals/patients were vaccinated with mRNA vaccines and healthcare professionals were vaccinated with AZ vaccines at the time of conduct of the study. The strengths of the study were however the quick response to the VITT focus with early blood sampling post-vaccination and the availability of pre-vaccination samples collected uniformly from the same individuals and participants in the ENFORCE vaccine trial. Furthermore, the head-to-head comparison of the AZ and mRNA vaccine response in the same study, minimizing variation is a clear strength. Finally, the inclusion of a broad range of both plasma and whole blood based analyses revealing inflammation, platelet activation and thrombin generation in the same study, is a clear strength.

In conclusion, the present study investigated the influence of different COVID-19 vaccines on inflammation, vascular endothelial activation, platelet activation and aggregation, whole blood coagulation, thrombin generation and PF4 antibodies to reveal potential differences between AZ and mRNA vaccines in individuals without VITT. The main findings were that the AZ vaccine induced a more pronounced increase in inflammation and platelet activation and higher thrombin generation compared to mRNA vaccines and that none of the vaccinated individuals developed PF4 antibodies. We speculate that specific components of the AZ adenovirus vector may serve as initial trigger(s) of (hyper)inflammation, platelet activation and thrombin generation, potentially lowering the threshold for a cascade of events that both trigger complications related to excessive inflammation, platelet and coagulation activation as observed in epidemiologic studies and promote development of VITT when combined with high-titer functionally active PF4 antibodies.

## Data Availability Statement

The raw data supporting the conclusions of this article will be made available by the authors, without undue reservation.

## Ethics Statement

The studies involving human participants were reviewed and approved by the National Ethical Committee (Denmark). The participants provided their written informed consent to participate in this study.

## Author Contributions

SO, OS, MT, NS, JL, LØ, and AH contributed to the design of the study. NS, OS, and AH participated in the enrolment of study participants and data collection. SO and AH led and supervised the laboratory analyses. SO and AH analyzed the data and drafted the manuscript. All authors contributed to the data interpretation and in revising the final manuscript. All authors contributed to the article and approved the submitted version.

## Conflict of Interest

The authors declare that the research was conducted in the absence of any commercial or financial relationships that could be construed as a potential conflict of interest.

## Publisher’s Note

All claims expressed in this article are solely those of the authors and do not necessarily represent those of their affiliated organizations, or those of the publisher, the editors and the reviewers. Any product that may be evaluated in this article, or claim that may be made by its manufacturer, is not guaranteed or endorsed by the publisher.
