# Supplementary material for: Inflammation and Platelet Activation After COVID-19 Vaccines - Possible Mechanisms Behind Vaccine-Induced Immune Thrombocytopenia and Thrombosis
Source: Front Immunol. 2021 Nov 23;12:779453. doi: 10.3389/fimmu.2021.779453 (PMC8649717; doi:10.3389/fimmu.2021.779453)
Supplement: Supplementary file 1 [file DataSheet_1.pdf]

Inflammation and platelet activation after COVID-19 vaccines - possible mechanisms behind Vaccine-induced Immune Thrombocytopenia and Thrombosis

Sisse Rye Ostrowski[1], Ole S. Søgaard[2], Martin Tolstrup[2], Nina B. Stærke[2], Jens Lundgren[3], Lars Østergaard[2], Anne-Mette Hvas[4]

[1]Dept. of Clinical Immunology, Copenhagen Hospital Biobank Unit, Rigshospitalet, University of Copenhagen and Dept. of Clinical Medicine, Faculty of Health and Medical Sciences, University of Copenhagen;
[2]Dept. Infectious Disease, Aarhus University Hospital and Dept. of Clinical Medicine, Aarhus University;
[3]Dept. of Infectious Diseases, Rigshospitalet, University of Copenhagen and Dept. of Clinical Medicine, Faculty of Health and Medical Sciences, University of Copenhagen;
[4]Dept. Clinical Biochemistry, Aarhus University Hospital, Aarhus University and Dept. of Clinical Medicine, Aarhus University

Correspondence
Anne-Mette Hvas, professor, chief physician, MD, PhD
Department of Clinical Biochemistry
Palle Juul-Jensens Boulevard 99
DK-8200 Denmark
am.hvas@dadlnet.dk

Supplementary

Table S1

Intercorrelations between platelet activation markers pre-, post-vaccination and delta values in all (n=80), AZ (n=55) and mRNA (n=25) vaccinated individuals.

Figure S1

Spearman correlations between post-vaccination platelet count and platelet activation markers (P-selectin, TGF-β and CD40L) pre- and post-vaccination (and delta values) with AZ (n=55) or mRNA (n=25) vaccines.

Table S1

Intercorrelations between platelet activation markers pre-, post-vaccination and delta values in all (n=80), AZ (n=55) and mRNA (n=25) vaccinated individuals.

		TGF-β Pre / Post / Delta	CD40L Pre / Post / Delta	TGF-β Pre / Post / Delta	CD40L Pre / Post / Delta	TGF-β Pre / Post / Delta	CD40L Pre / Post / Delta
		All n=80		AZ n=55		mRNA n=25	
P-selectin	Pre	rho=0.738 p<0.0001	rho=0.698 p<0.0001	rho=0.665 p<0.0001	rho=0.627 p<0.0001	rho=0.819 p<0.0001	rho=0.758 p<0.0001
	Post	rho=0.374 0.001	rho=0.490 p<0.0001	rho=0.415 0.002	rho=0.489 p<0.0001	rho=0.531 0.006	rho=0.482 0.015
	Delta	rho=0.402 p<0.0001	rho=0.487 p<0.0001	rho=0.445 0.001	rho=0.495 p<0.0001	rho=0.468 0.018	rho=0.495 0.012
TGF-β	Pre	N/A	rho=0.944 p<0.0001	N/A	rho=0.944 p<0.0001	N/A	rho=0.926 p<0.0001
	Post	N/A	rho=0.688 p<0.0001	N/A	rho=0.725 p<0.0001	N/A	rho=0.897 p<0.0001
	Delta	N/A	rho=0.792 p<0.0001	N/A	rho=0.734 p<0.0001	N/A	rho=0.899 p<0.0001

Correlations between post-vaccination platelet count and pre-, post-vaccination and delta-value platelet activation marker levels were assessed by Spearman correlations displaying rho and p-values. Only the same time-points were compared i.e. pre- vs pre-vaccination, post- vs post-vaccination and delta vs delta values.

Figure S1

Spearman correlations between post-vaccination platelet count and platelet activation markers (P-selectin, TGF-β and CD40L) pre- and post-vaccination (and delta values) with AZ (n=55) or mRNA (n=25) vaccines. AZ=blue dots. mRNA=gray dots.

P-selectin

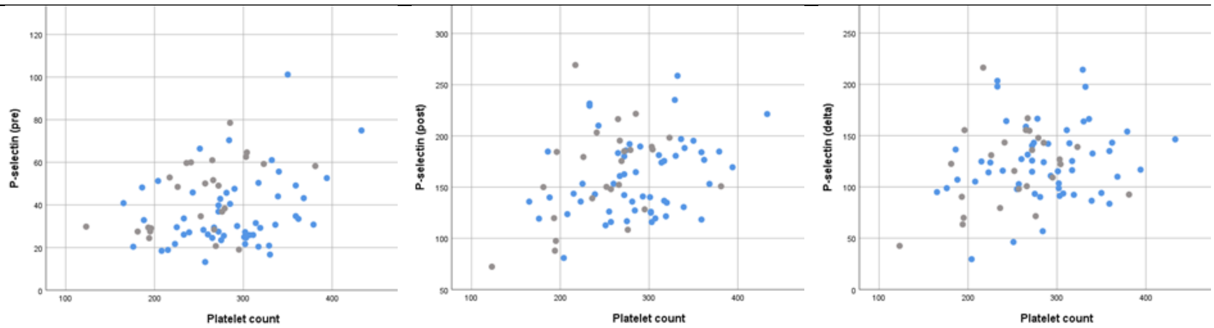

Pre	Post	Delta
All: rho=0.215, p=0.057	All: rho=0.246, p=0.028	All: rho=0.149, p=NS
AZ: rho=0.268, p=0.050	AZ: rho=0.257, p=0.058	AZ: rho=0.137, p=NS
mRNA: rho=0.445, p=0.026	mRNA: rho=0.428, p=0.033	mRNA: rho=0.234, p=NS

TGF-β

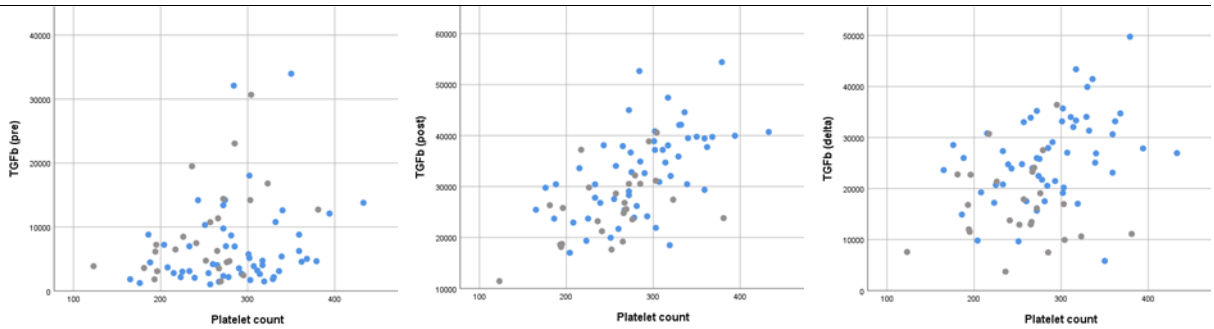

Pre	Post	Delta
All: rho=0.221, p=0.051	All: rho=0.603, p<0.0001	All: rho=0.365, p=0.001
AZ: rho=0.253, p=0.065	AZ: rho=0.604, p<0.0001	AZ: rho=0.416, p=0.002
mRNA: rho=0.431, p=0.032	mRNA: rho=0.562, p=0.003	mRNA: rho=-0.042, p=NS

CD40L

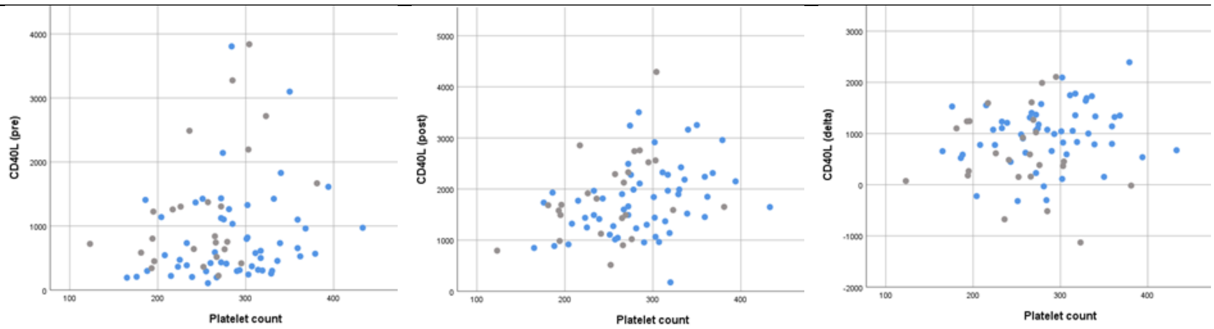

Pre	Post	Delta
All: rho=0.214, p=0.058	All: rho=0.374, p=0.001	All: rho=0.163, p=NS
AZ: rho=0.272, p=0.046	AZ: rho=0.420, p=0.001	AZ: rho=0.226, p=NS
mRNA: rho=0.409, p=0.042	mRNA: rho=0.403, p=0.046	mRNA: rho=-0.108, p=NS

Inter correlations between platelet activations markers pre-, post-vaccination and delta-value levels were assessed by Spearman correlations displaying rho and p-values.
